# Characteristics of Social Network Gamers: Results of an Online Survey

**DOI:** 10.3389/fpsyt.2015.00069

**Published:** 2015-07-08

**Authors:** Olga Geisel, Patricia Panneck, Anna Stickel, Michael Schneider, Christian A. Müller

**Affiliations:** ^1^Department of Psychiatry, Campus Charité Mitte, Charité – Universitätsmedizin Berlin, Berlin, Germany

**Keywords:** Internet addiction, Internet use disorder, behavioral addiction, social networking sites, online role-playing games, alexithymia

## Abstract

Current research on Internet addiction (IA) reported moderate to high prevalence rates of IA and comorbid psychiatric symptoms in users of social networking sites (SNS) and online role-playing games. The aim of this study was to characterize adult users of an Internet multiplayer strategy game within a SNS. Therefore, we conducted an exploratory study using an online survey to assess sociodemographic variables, psychopathology, and the rate of IA in a sample of adult social network gamers by Young’s Internet Addiction Test (IAT), the Toronto Alexithymia Scale (TAS-26), the Beck Depression Inventory-II (BDI-II), the Symptom Checklist-90-R (SCL-90-R), and the WHO Quality of Life-BREF (WHOQOL-BREF). All participants were listed gamers of “Combat Zone” in the SNS “Facebook.” In this sample, 16.2% of the participants were categorized as subjects with IA and 19.5% fulfilled the criteria for alexithymia. Comparing study participants with and without IA, the IA group had significantly more subjects with alexithymia, reported more depressive symptoms, and showed poorer quality of life. These findings suggest that social network gaming might also be associated with maladaptive patterns of Internet use. Furthermore, a relationship between IA, alexithymia, and depressive symptoms was found that needs to be elucidated by future studies.

## Introduction

In the last decade, the number of Internet users worldwide increased from 12.3/100 people to 32.8 (1). Similarly, the use of so-called social networking sites (SNS) increased continuously during the last years. SNS mainly contain individual user profiles that are linked to those of other users electronically. Currently, the SNS “Facebook” represents one of the most widely used sites with >1 billion monthly active users and >600 million daily active users (2). Although SNS usage is part of today’s daily routine for many people worldwide and even benefits for children and adolescents (i.e., enhancing communication, social, or technical skills) were reported by few authors (3), it also might be one field with a putative high prevalence of addictive behavior, i.e., Internet addiction (IA) (4–6).

The term “Internet addiction” refers to a condition characterized by the inability to control Internet use, potentially resulting in social, academic, occupational, and financial impairments (7). At present, there is no consensus on how diagnostic criteria of IA should be defined and IA is not yet included into the ICD-10 (8). In 2013, the American Psychiatric Association (APA) included “Internet gaming disorder” (IGD) in section III of DSM-V (9), a section dedicated to conditions that require further research. However, IA is a heterogeneous disorder category with several subtypes besides online gaming (e.g., social networking, messaging, online sexual pre-occupations) (7, 10) and diagnostic tools to precisely assess IA are still lacking.

Several self-report questionnaires have been developed to describe problematic use of the Internet – for example, the Young Internet Addiction Test (IAT) (7). To assess different subtypes of IA, questionnaires for specific forms of Internet use have also been developed (11).

In recent years, numerous online gaming applications designed for the use within a SNS have been released. To our knowledge, research about the population using those games frequently is scarce and current findings are inconsistent. Research on SNS users and Internet gamers provided differing prevalence rates of IA. Smahel and co-workers reported that about 40% of massively multiplayer online role-playing games (MMORPGs) users of their sample categorized themselves as “addicted to the game” (12). By contrast, a study in college students using SNS found that one in six of the study participants reported frequent problems in life due to “Facebook” use (6).

IA has also been reported to be often accompanied by other psychiatric symptoms and difficulties in daily life functioning (7). Some studies reported a high rate of depressive symptoms in subjects with IA (13–15), whereas other research groups could not find a relationship between problematic Internet use and depression (16).

Beyond depression, the concept of alexithymia might be relevant regarding the development and maintenance of IA. According to Nemiah et al., alexithymic individuals have difficulties in identifying and describing their emotions, can hardly distinguish between feelings and bodily sensations caused by emotional arousal, and show externally oriented thinking (17). Alexithymia was reported to be common among individuals with substance use disorders (18) and may increase the risk for IA (19). De Berardis and co-workers found that alexithymic individuals in a non-clinical sample of undergraduate college students reported more excessive Internet use and showed higher scores in the IAT. Compared to non-alexithymic individuals, significantly more alexithymics fulfilled criteria of IA in their study (24.2% alexithymics vs. 3.2% non-alexithymics). Furthermore, a recent study found that the severity of IA was positively correlated with alexithymia in a sample of Turkish college students (20). Also, Scimeca et al. found that there was a correlation between levels of alexithymia and IA, and that alexithymia even qualified as a predictor of IA scores (21). In line with those findings, Kandri et al. (22), who took sociodemographic as well as emotional profiles of Internet users into account, found that alexithymia and excessive Internet use were strongly related.

Our study aimed to characterize the subgroup of social network gamers with respect to sociodemographic variables, psychopathology, and the rate of IA. We exemplarily focused on users of the game “Combat Zone” offered by the social networking site “Facebook.”

## Materials and Methods

We contacted a “Facebook” game provider to recruit adults for an online survey. All participants of this study were listed gamers of “Combat Zone” in “Facebook” and received an invitation to participate in our study via “Facebook.” “Combat Zone” is a multiplayer strategy game that can only be played while logged into “Facebook.” The participant’s account data are used to create an avatar that is capable of military strikes. The gamers buy or sell territory, form alliances, or fight enemies by selecting options proposed by the provider. No special visual effects are used and the game is meant to be played slowly, while communicating with other users on “Facebook” (23).

Once participants connected to our website, they had access to informations on the researchers, aims of the study and clear instructions on the questionnaires and their right to withdraw from the study at any time. The participants were asked to accept the invitation to complete an online survey. After obtaining this online informed consent, participants could complete the survey at any time or withdraw from the study at any timepoint. Questionnaires were strictly anonymous and no data regarding the identity of the participants were collected. Subjects who completed the survey received profit in the form of game boni from the provider. For inclusion in this study, participants had to be older than 18 years and had to use their SNS account very frequently (i.e., daily use for a minimum of 1 h during the last 3 months). The study was approved by the local ethics committee and adhered to the principles of the Declaration of Helsinki. Informed consent was obtained from all participants as described above.

Our measures contained the IAT, a validated screening instrument for problematic use of the Internet (7, 24). Its 20 questions assess the degree to which Internet use affects daily routines, social life, occupation, sleep, or emotions and are rated on a 6-point frequency scale and summed. According to previous studies (15, 25, 26), an IAT score of ≥50 was defined as IA.

Furthermore, we used the Toronto Alexithymia Scale (TAS-26) (27), which was developed as a standardized self-assessment questionnaire to measure alexithymia. It consists of 26 items that are rated on a 5-point Likert scale and result in three scales: (1) difficulty in identifying feelings, (2) difficulty in describing feelings, and (3) externally oriented thinking. These scales are summed up to a total score. The Beck Depression Inventory-II (BDI-II) (28) and the Symptom Checklist SCL-90-R (29) were used to explore depressive and other psychiatric symptoms. The BDI-II is a 21-items self-questionnaire and used to measure severity of depressive symptoms. Psychological and physiological symptoms of depression are rated on a 0–3 scale and summed. The SCL-90-R consists of 90 items that are rated on a 5-point scale ranging from “not at all” to “extremely.” The items cover nine domains (somatization, obsessive–compulsive thoughts, interpersonal sensitivity, depression, anxiety, hostility, phobic anxiety, paranoid conceiving, and psychotic behavior), and a general severity index (GSI), indicating the overall psychological distress. The results of the SCL-90-R are given in *T* values, a value of ≥60 is considered as above average (mean = 50, SD = 10).

Finally, the participants’ quality of life was assessed using the short version of the World Health Organization Quality of Life Measurement (WHOQOL-BREF) (30). Twenty-six items are rated on a scale ranging from 1 to 5. The four domain scores physical, psychological, social, and environment can be derived and illustrate different aspects of quality of life. The scores are transformed on a scale from 0 to 100 with higher scores indicating a higher quality of life.

### Statistical analyses

Results are presented as mean ± SD. The Kolmogorov–Smirnov test was used to assess normal distribution. Due to non-normal distributions only non-parametric statistics were applied; differences between participants with and without IA were analyzed using the Mann–Whitney *U* test. Rank correlation coefficients (Spearman’s ρ) were calculated for sociodemographic and clinical variables. The chosen level of significance was *p* < 0.05. Statistical analyses were performed using IBM SPSS Statistics version 19 (SPSS Inc., Chicago, IL, USA).

## Results

### Subjects

Five hundred twenty-eight subjects connected to our website. However, 158 subjects had to be excluded from the study due to missing and/or inconsistent data. Thus, 356 male and 14 female subjects were included in the final analysis (*n* = 370, 70.1%). Sociodemographic characteristics of the study population are listed in Tables 1 and 2.

**Table 1:** **Sociodemographic characteristics of the study participants I**.

	Mean	SD
Age (years)	38.9	13.4
Height (m)	1.78	0.12
Weight (kg)	91.0	22.6
BMI (kg/m^2^)	28.7	7.2
Number of children	1.6	1.62
Number of individuals in household	3.05	1.53

**Table 2:** **Sociodemographic characteristics of the study participants II**.

	Total of subjects (*n*)	Percentage
Gender
Male	356	96
Female	14	4
Marital status
Single	122	33
In a relationship	248	67
Geographical localization
USA	296	80
UK	46	12
Canada	10	3
Australia	7	2
Germany	6	2
Other	5	2
Educational level
Below high school diploma	89	24
High school diploma to university degree	191	52
Masters degree and higher	90	24
Occupation
None	115	31
Student	25	7
Working	230	62

In the IAT data analysis, 16.2% of the participants (*n* = 60) were categorized as subjects with IA (total score ≥50). Furthermore, 13.3% of these participants (*n* = 8) had severe problems with Internet use according to Young (total score ≥80) (31). None of the 60 subjects with IA was female.

Using a cut-off score of 54 in the TAS-26 (27), 19.5% (*n* = 72) of the participants in our study fulfilled the criteria for alexithymia.

BDI-II data analysis revealed that 76.5% (*n* = 283) of the participants had no or minimal depressive symptoms (score <14), 10% (*n* = 37) showed mild symptoms (score of 14–19), 7.0% (*n* = 26) showed moderate symptoms (score of 20–28), and 6.5% (*n* = 24) showed severe symptoms of depression (score of 29–63).

The SCL-90 GSI did not reveal increased levels of psychiatric symptoms in the analysis of all subjects (mean = 52.0, SD = 19.1). The WHOQOL-BREF for all subjects (*n* = 370) did not show a reduced quality of life (physical health: mean = 69.3, SD = 19.7; psychological: mean = 70.1, SD = 20.8; social relationships: mean = 62.8, SD = 23.8; environment: mean = 67.0, SD = 19.7).

Severity of IA was positively correlated with the SCL-90-R GSI score (*r* = 0.136, *p* = 0.009). Also, severity of IA was positively correlated with BDI-II total scores (*r* = 0.210, *p* = 0.000). There was a negative correlation between severity of IA and WHOQOL-BREF scores (physical health: *r* = −0.277, *p* = 0.000; psychological: *r* = −0.329, *p* = 0.000; social relationships: *r* = −0.257, *p* = 0.000, environment: *r* = −0.198, *p* = 0.000).

A positive correlation was found for the TAS-26 subscale “externally oriented thinking” and severity of IA (*r* = 0.114, *p* = 0.028).

The mean BMI in our sample was 28.7 kg/m^2^ (SD = 7.2). Thirty-six percent of the participants (*n* = 133) reported to be overweight (BMI 25–29.99 kg/m^2^), 23% (*n* = 85) were obese class I (BMI 30–34.99 kg/m^2^), and 13% (*n* = 47) obese class II or III (BMI ≥35 kg/m^2^) (32). Twenty-six percent of the participants (*n* = 98) reported normal weight to mild thinness (BMI 17–24.99 kg/m^2^), and 2% (*n* = 6) reported a BMI <17 kg/m^2^, indicating moderate to severe underweight. BMI was positively correlated with age of participants (*r* = 0.328, *p* = 0.000), but did not correlate with any clinical variable.

### Comparison of subjects with and without IA

Significant differences in the TAS-26, BDI-II, and WHOQOL-BREF questionnaires were found comparing subjects with IA (*n* = 60) and participants without IA (*n* = 310, see Table 3). The IA group had significantly more subjects with alexithymia (*Z* = −2.606, *p* = 0.009), reported more depressive symptoms (*Z* = −2.438, *p* = 0.015), and showed poorer quality of life (physical health: *Z* = −4.455, *p* = 0.000; psychological: *Z* = −5.139, *p* = 0.000, social relationships: *Z* = −3.679, *p* = 0.000, environment: *Z* = −2.561, *p* = 0.010). There were no significant differences in sociodemographic characteristics or SCL-90-R scales between both groups.

**Table 3:** **Comparison of subjects with and without IA**.

	Subjects with IA	Subjects without IA	Statistical analysis of significance by Mann–Whitney *U* test
Age (mean ± SD)	36.5 ± 14.6	39.4 ± 13.4	*Z* = −1,504, *p* = 0.132
Children in household (mean ± SD)	1.5 ± 1.6	1.6 ± 1.6	*Z* = −0.814, *p* = 0.415
Persons in household (mean ± SD)	3.1 ± 1.6	3.0 ± 1.5	*Z* = −0.409, *p* = 0.683
BMI (kg/m^2^) (mean ± SD)	27.4 ± 6.8	29.0 ± 7.3	*Z* = −1.925, *p* = 0.054
BMI <25 (*n*/%)	19/32.2	85/27.4	
BMI 25–29.99 (*n*/%)	24/40.7	109/35.2	
BMI 30–34.99 (*n*/%)	8/13.6	77/24.8	
BMI ≥35 (*n*/%)	8/13.6	39/12.6	
TAS-26 (score ≥54) (*n*/%)	19/31.7	53/17.1	*Z* = −2.606, *p* = 0.009
Difficulty in identifying feelings	15.5 ± 6.6	15.1 ± 5.5	*Z* = −0.335, *p* = 0.737
Difficulty in describing feelings	13.2 ± 3.4	12.8 ± 3.9	*Z* = −0.977, *p* = 0.329
Externally oriented thinking	16.6 ± 5.2	15.1 ± 4.2	*Z* = −2.271, *p* = 0.023
SCL-90 (GSI) (mean ± SD)	54.5 ± 20.5	51.5 ± 18.9	*Z* = −1.025, *p* = 0.305
BDI (total score) (mean ± SD)	11.9 ± 11.7	7.5 ± 10.0	*Z* = −2.438, *p* = 0.015
BDI severity (*n*/%)			
Minimal: 0–13	37/61.7	247/79.7	*Z* = −3.436, *p* = 0.001
Mild: 14–19	8/13.3	29/9.4	
Moderate: 20–28	8/13.3	18/5.8	
Severe: 29–63	7/11.7	16/5.2	
WHOQOL-BREF (Mean ± SD)			
Physical health	59.7 ± 19.0	71.2 ± 19.3	*Z* = −4.455, *p* = 0.000
Psychological	59.0 ± 17.7	72.3 ± 20.7	*Z* = −5.139, *p* = 0.000
Social relationships	53.6 ± 21.6	64.6 ± 23.9	*Z* = −3.679, *p* = 0.000
Environment	62.6 ± 16.8	67.8 ± 20.2	Z = −2.561, *p* = 0.010

*BMI, body mass index; *p*, level of significance for Mann–Whitney *U* test; *Z*, statistical parameter of *U* test*.

## Discussion

The present study explored the characteristics of SNS gamers by online self-report questionnaires, focusing on the rate of IA, alexithymia, and further psychiatric symptoms. In this sample, 16% of the participants reached the cut-off score of 50 in the IAT, representing participants who experience occasional or frequent problems due to Internet use (31). By contrast, a huge American online survey with 17,251 participants reported a distinctly lower prevalence of IA of approximately 6% (33). Of course, since sample sizes and study designs vary substantially, a direct comparison is only of limited value. However, in line with our findings, a recent study in Turkish university students using SNS reported that 12.2% of the participants were categorized as “Internet addicted” or at “high risk for addiction” according to the Internet Addiction Scale (IAS) (20). Studies on prevalence of IA in MMORPGs users revealed even higher rates of problematic Internet use within this population. In a recent study, 44.2 and 32.6% of a sample of MMROPGs users were categorized as subjects with IA as assessed by the Goldberg Internet Addiction Disorder scale (GIAD) and the Orman Internet Stress Scale (ISS), respectively (34). Taken together, prevalence rates found in these studies differed substantially, possibly related to different age groups, Internet user subtypes and especially different diagnostic tools to assess IA.

The very small proportion of women of 3.8% in our sample might possibly result from the chosen application. According to the provider of “Combat Zone,” mean percentage of female gamers was around 4% in the last 2 years. The fact that none of the female gamers was categorized as subject with IA is a phenomenon, which has already been observed in previous studies; possibly, male gamers might be more susceptible to IA (35).

Our results are in line with previous reports of a relationship between alexithymia and IA (18, 19), but we explored a specific subgroup of Internet usage. There was a significantly higher rate of alexithymia in subjects with IA compared to those participants without IA (31.7 vs. 17.1%). The severity of IA was positively correlated with the “externally oriented thinking” subscale of the TAS-26. Yet, it remains unclear whether alexithymia predisposes for IA. One might speculate that alexithymic individuals tend to use the Internet more excessively as a result of lower self-esteem (36) and the possibility to avoid “real” social interactions, as proposed previously (19).

The current study also confirms the results of previous research that links problematic Internet use to higher levels of depression (14, 15, 20, 37). One assumption might be that patients with depression possibly try to alleviate different symptoms by excessive use of social network games. On the other hand, pathological patterns of Internet use might also evoke depressive symptoms (38). Therefore, future studies are needed to elucidate the precise relationship between IA and depression.

It is interesting to note that approximately three out of four participants were overweight or obese. However, overweight/obesity was not related to any clinical variable in this study. Thus, these findings need to be investigated in further studies.

Our results suggest that patients with IA should be carefully screened for relevant comorbidities such as depressive disorders, alexithymia, and eating disorders. Regarding the treatment of IA, particularly cognitive–behavorial therapy might represent a promising treatment approach (36).

Several limitations of this study restrict the interpretation of the results. First, the gender distribution was highly unbalanced in the present study. Second, our sample was drawn from only one “Facebook” application and therefore does obviously not represent all types of Internet users, diminishing the external validity of the results. Furthermore, the sample size of this study was too small to draw clear conclusions. Moreover, the self report measures used are susceptible to bias, as seen in the rate of excluded data. A clinical interview with additional data from external informants such as family members might have provided more reliable data. Finally, the lack of standardized clinical instruments to assess IA might have influenced the outcome of the study.

## Conclusion

We found that nearly one in six SNS gamers met the criteria for IA in our sample. Comparing study participants with and without IA, the IA group had more subjects with alexithymia, reported more depressive symptoms, and showed poorer quality of life. These findings suggest that social network gaming might also be associated with maladaptive patterns of Internet use. Furthermore, a relationship between IA, alexithymia, and depressive symptoms was found that needs to be elucidated by future studies.

## Conflict of Interest Statement

The authors declare that the research was conducted in the absence of any commercial or financial relationships that could be construed as a potential conflict of interest.
